# Hot pack therapy versus cherry seed pillow in fibromyalgia patients: a prospective randomized controlled trial

**DOI:** 10.1590/1806-9282.20250365

**Published:** 2025-09-19

**Authors:** Nilay Bektas Akpinar, Hasan Gercek, Ulviye Özcan Yüce, Sinan Bagcaci

**Affiliations:** 1 Ankara Medipol University, Faculty of Health Sciences, Department of Nursing – Ankara, Turkey.; 2 KTO Karatay University, Vocational School of Health Services, Department of Physiotherapy – Konya, Turkey.; 3 Osmaniye Korkut Ata University, Faculty of Health Sciences, Department of Nursing – Osmaniye, Turkey.; 4 KTO Karatay University, Department of Physiotherapy and Rehabilitation – Konya, Turkey.

**Keywords:** Cherry, Fibromyalgia, Pain, Physalis, Quality of life

## Abstract

**OBJECTIVE:**

The aim of this study was to evaluate the effect of a cherry seed pillow on pain and quality of life in fibromyalgia patients.

**METHODS:**

This study used a pretest-posttest, randomized, and controlled prospective research design. It was conducted between December 2023 and February 2024 in the physiotherapy clinic of a private hospital. A total of 72 adults with fibromyalgia were randomly assigned to the cherry seed (n=36) and control (n=36) groups. Both groups received conventional physiotherapy 5 days a week for 4 weeks. A cherry seed pillow (microwave-heated) was used in the cherry seed group. A hot pack was applied to the control group. The data were collected using a personal information form, Visual Analog Scale, and Fibromyalgia Impact Questionnaire.

**RESULTS:**

The change in the participants’ resting pain before and after the treatment decreased in both the treatment (p<0.001, d=0.86) and control (p<0.001, d=0.97) groups. The change in activity pain decreased in the intervention (p<0.001, d=0.79) and control (p<0.001, d=0.87) groups. Similarly, there was a decrease in night pain in the intervention (p<0.001, d=0.86) and control (p<0.001, d=0.89) groups. The change in Fibromyalgia Impact Questionnaire scores of the participants showed a decrease in the intervention (p<0.001, d=3.90) and control (p<0.001, 30.03, d=4.28) groups.

**CONCLUSION:**

Both groups showed a reduction in pain, likely due to the combined effect of heat and physiotherapy; however, the clinical improvement was more significant in the control group, suggesting that the cherry seed pillow was less effective.

## INTRODUCTION

Fibromyalgia is a chronic musculoskeletal illness characterized by widespread body pain of uncertain etiology^1,2^. Fibromyalgia is the third most frequent rheumatic disease, and its increase is in women, those with lower socioeconomic status, and those in the 50–60 age range^2,3^.

Fibromyalgia affects individuals physically, psychologically, and socially negatively, causing difficulties in performing daily activities and decreased quality of life^2,4,5^. The aim of the treatments to be applied is to improve the physical functions of the patients and to improve their general health and quality of life. Therefore, pain reduction is the first step in treatment^3^. It is recommended that the treatment to be applied should be patient-oriented^2^. In general, pharmacologic and non-pharmacologic methods are recommended to be applied together in a holistic treatment approach^2,3-7^. Pharmacologic treatment includes analgesics, antipyretics, muscle relaxants, etc^2,7,8^. Non-pharmacologic treatment methods include education, acupuncture, exercise, and physiotherapy options such as hot applications^2,4,6-11^. In fact, it has been reported that almost all fibromyalgia patients use some form of non-pharmacological methods like prayer, wet cupping, herbal therapy for pain, and other symptoms^4,7^. One of the common applications used for this purpose is heat application. Regional heat applications are used to increase the elasticity of connective tissue, reduce muscle spasms, and reduce pain^10^. The most preferred method of regional heat application is hot pack application^12^.

Cherry seed has a high content of phenolic compounds and are used to support and reduce symptoms in some disorders due to their antimicrobial and anti-inflammatory properties^13^. Cherry seed pillows are used to relieve various types of pain, including childbirth, cancer, kidney stones, and infant colic pain^14-16^.

As far as we are aware from the literature, it has been reported that methods such as hot pack, hydrotherapy, and whirlpool have been used as heat agents in individuals with fibromyalgia, but cherry seed application has not been encountered^12,17,18^. Since cherry seed pillow is preferred by patients in our country for pain reduction, has no side effects, is easy and a pillow can be applied repeatedly for the same patient, this study was aimed to evaluate the effect of cherry seed pillow on pain and quality of life in fibromyalgia patients in addition to standard medical treatment.

## METHODS

This study used a pre-posttest, randomized, and controlled prospective research design.

### Participants

**Figure 1:**
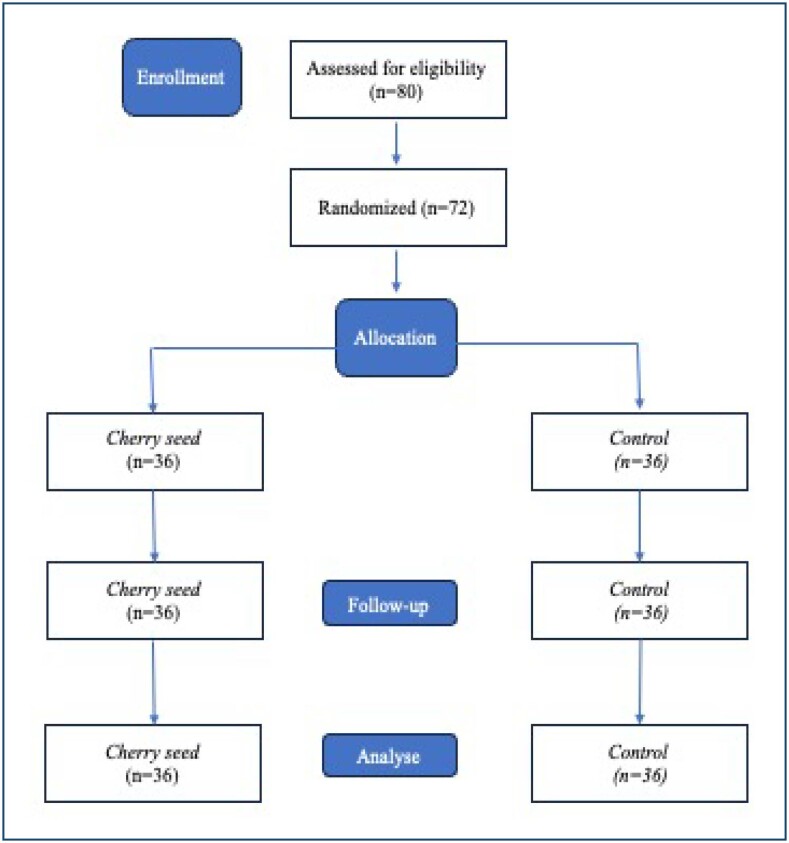
The CONSORT chart of the study.

This study was carried out in a private hospital's physiotherapy clinic between December 2023 and February 2024. A total of 72 volunteers with fibromyalgia were included in the study (Figure 1).

The requirements for inclusion were (1) having a fibromyalgia diagnosis based on the 2010 categorization criteria of the American College of Rheumatology (ACR) 2010, having a normal neuromuscular system examination, having a pain (VAS-Visual Analog Scale) level of at least five points, having no communication problems, being willing to participate in the practice of using the cherry seed pillow, being able to answer the data collection tools to be used in the study, and not using hot, cold, etc. applications at home.

Exclusion criteria: Communication problems, not accepting the application of using the cherry seed pillow, and not completing the application process, having a musculoskeletal system disorder other than fibromyalgia, being pregnant, or breastfeeding.

### Randomization and blinding

Participants were randomly assigned to either the cherry seed group or the control group in a 1:1 allocation ratio using a computer-generated random sequence via an internet-based randomization tool (www.randomizer.org). The randomization process was carried out by an independent investigator who was not involved in the recruitment or evaluation of participants. Allocation concealment was ensured through this procedure, as the group assignments were not known in advance by the researchers responsible for enrolling participants. Each participant was informed that they would be randomly assigned to one of the two study groups. Outcome assessments were performed by a blinded investigator at both baseline and immediately after the intervention.

### Sample size

The G*Power software (version 3.0.10; Franz Foul, Universitat Kiel, Germany) was used to determine the sample for the study. The size of the sample was calculated as at least 36 individuals in each group for the effect size of 0.389, Type I error of 0.05, and power of 90% obtained as 10 participants who were involved in our pilot study^5,6^.

### Cherry seed and the control group

The paraspinal muscles of the cherry seed and both group participants were treated bilaterally with transcutaneous electrical nerve stimulation (TENS; Chattanooga Inc., Hixson, TN, USA) for 20 min at a frequency of 100 Hz and ultrasound (Chattanooga, TN, USA) for 5 min at an intensity of 1.5 W/cm^2^, a frequency of 1 MHz, and continuous mode, 5 days a week for 4 weeks. In addition to physiotherapy interventions, all participants were given a home program consisting of 30 min of moderate-intensity walking, low-to-medium-resistance posture exercises, neck range of motion, and shoulder-strengthening exercises with a light resistance band 3 days a week. The physiotherapist responsible for all physiotherapy applications had 10 years of experience. A cherry pit pillow (20×50 cm) was heated in a microwave oven at 500 W for 5 min prior to application. A microwave-heated pillow filled with cherry seeds was applied to the patient's back for 20 min. Control group: the patient had a hot pack applied to their back for 20 min.

### Data collection

The data were collected using a personal information form, VAS, and Fibromyalgia Impact Questionnaire (FIQ). A blinded evaluator conducted all measurements before and at the end of the study. The blinded evaluator in the first intervention carried out the personal information form, VAS, and FIQ to all patients face to face. At the final evaluation of all the patients, FIQ as well as VAS values were recorded at night, at rest, and while active. In addition, they will be evaluated again in both groups.

### Personal information form

A literature review^4-6^ was used to develop the personal information form. The personal information form contains questions regarding demographic characteristics. In addition, there are two inquiries: diagnosis and frequency of pain.

### Visual Analog Scale

The scale has a range of 0 to 10, where a score of "0" indicates no pain and a score of "10" indicates unbearable pain. VAS was utilized to determine the extent of pain during rest, activity, and sleep. The participants were compelled to assess their pain scores both before and after 4 weeks of treatment^19^.

### Fibromyalgia Impact Questionnaire

The FIQ was used to assess the quality of life of participants. The FIQ is a 10-question scale that measures how much a person is affected by the symptoms of fibromyalgia syndrome (FMS). The FIQ takes into consideration 10 different characteristics: physical function, well-being, absenteeism from work, difficulty at work, pain, fatigue, morning fatigue, stiffness, anxiety, and depression. The total score that can be obtained from the questions is between a minimum of 0 and a maximum of 80 points. A higher score means that the person is more affected by FMS^20^.

### Statistical analysis

The data were evaluated by using the Statistical Package for the Social Sciences software version 29 (IBM Corp., Armonk, NY, USA). For categorization and continuous variables, descriptive statistics (mean, standard deviation, number, and percentile) were provided. The Shapiro-Wilk test and histogram method were utilized to evaluate the consistency of the variables with normal distribution. To compare differences before and after the application, a paired sample t-test was utilized because the data were in line with a normal distribution. The statistical significance level was accepted as p<0.05.

### Ethics

This study was prospectively registered on clinicaltrials.gov (NCT 06171022), and Ethics Committee approval for a randomized clinical trial was obtained from Ankara Medipol University University Ethics Committee (no:150). Before the study began, participants were informed about it and given informed consent.

## RESULTS

Sociodemographic data of the participants are given in Table 1.

**Table 1:** Participants’ sociodemographic data.

		Cherry seed (n=36) X±SD/n (%)	Control (n=36) X±SD/n (%)	p
Age (year)		39.30±6.28	41.61±4.91	0.087^a^
BMI (kg/m^2^)		27.32±4.79	28.25±1.26	0.271^a^
Fibromyalgia duration (month)		24.58±11.74	27.17±11.41	0.347^a^
**Gender**				
	Female	27 (75.0%)	25 (69.4%)	0.792^b^
	Male	9 (25.0%)	11 (30.6%)	
**Marital status**				
	Single	4 (11.1%)	2 (5.6%)	0.693^b^
	Married	29 (80.6%)	31 (86.1%)	
	Divorced	3 (8.3%)	3 (8.3%)	
**Employe status**				
	Employed	27 (75.0%)	29 (80.6%)	0.300^b^
	Unemployed	8 (22.2%)	4 (11.1%)	
	Retired	1 (2.8%)	3 (8.3%)	
**Income status**				
	Income is less than expenditure	7 (19.4%)	9 (25.0%)	0.765^b^
	Income is equal to expenditure	22 (61.2%)	19 (52.8%)	
	Income is more than expenditure	7 (19.4%)	8 (22.2%)	
**Pain frequency**				
	0–3 months	26 (72.2%)	29 (80.6%)	0.652^b^
	3–6 months	8 (22.2%)	5 (13.8%)	
	6–9 months	2 (5.6%)	2 (5.6%)	

n: number; X: mean; SD: standard deviation; %: percentage; BMI: body mass index;

a independent sample t-test;

b Chi-square; p<0.05.

Participants had similar rest pain before the study (Table 2). The change in the participants’ resting pain before and after the treatment decreased in both the treatment (p<0.001, 95%CI 3.13, 3.71, d=0.86) and control (p<0.001, 95%CI 3.49, 4.15, d=0.97) groups. The change in activity pain decreased in the intervention (p<0.001, 95%CI 3.04, 3.58, d=0.79) and control (p<0.001, 95%CI 3.68, 4.27, d=0.87) groups. Similarly, there was a decrease in night pain in the intervention (p<0.001, 95%CI 1.88, 2.46, d=0.86) and control (p<0.001, 95%CI 2.93, 3.54, d=0.89) groups (Table 2). Although both interventions were effective for rest, activity, and night pain, the effect size was greater in the control group. In addition, night pain was significantly reduced in the control group between posttests (p<0.001).

**Table 2:** Participants’ baseline, pre- and post-interventional pain and quality of life scores.

VAS	Groups	Before X±SD	After X±SD	p
VAS at rest	Cherry seed	6.93±1.15	3.31±1.09	**<0.001** b
	Control	7.32±0.89	3.51±1.13	**<0.001** b
	p	0.110^a^	0.987	
VAS at activity	Cherry seed	7.33±0.91	4.01±0.98	**<0.001** b
	Control	7.62±0.77	3.64±0.99	**<0.001** b
	p	0.144	0.118	
VAS at night	Cherry seed	5.54±0.81	3.36±0.89	**<0.001** b
	Control	5.81±0.46	2.58±0.74	**<0.001** b
	p	0.081	**<0.001** a	
FIQ	Cherry seed	53.52±10.37	30.69±7.83	**<0.001** b
	Control	56.28±8.88	27.97±6.86	**<0.001** b
	p	0.231	0.121	

n: number; X: mean; SD: standard deviation; VAS: Visual Analog Scale; FIQ: Fibromyalgia İmpact Questionnaire;

a Independent sample t-test;

b Paired sample t-test;

p<0.05. Statistically significant results (p<0.05) are presented in bold.

Participants had similar FIQ scores (p=0.231) before the study (Table 2). The change in FIQ scores of the participants showed a decrease in the intervention (p<0.001, 95%CI 21.51–24.15, d=3.90) and control (p<0.001, 95%CI 27.13–30.03, d=4.28) groups (Table 2).

## DISCUSSION

In our study, pain levels decreased in both groups. However, we can say that a cherry seed pillow is not as effective as a hot pack in reducing pain. There may be several reasons for this. Although cherry pits absorb heat quickly, they may not retain it as long as hot packs. In addition, hot packs are more effective in delivering heat to deeper tissues due to their thermal conductivity^21,22^. However, other studies in the literature have found that hot pack and other physiotherapy applications are effective in fibromyalgia patients, similar to our research results^17,18,21^. Physiotherapy interventions (such as exercise, manual therapy, and TENS) showed positive medium- and long-term effects on pain and quality of life in individuals with fibromyalgia^18^.

We were unable to find any studies involving cherry seed application in fibromyalgia in the literature^7,9,23^. However, there were studies in which the effects of flaxseed pillow and mustard seed pillow on preterm infants were examined^24,25^. Although the effect of the ingredients varies, these are the studies that we can interpret most closely. Flaxseed pillow treatment was applied to premature infants for 5 days, and it was found that stress levels of this group decreased compared to the control group^24^. A group of infants born ≤32 weeks and <1,500 g admitted to the neonatal intensive care unit was treated with a mustard seed pillow. This group had lower Cranial Vault Asymmetry Index (CVAI) scores than the control group^24^. Previous studies have found that fibromyalgia patients prefer warming as the second method to reduce pain^19^. In addition, warm applications are thought to reduce pain^18,26,27^. By heating the cherry seed pillow, the pain-reducing effect of the heat is utilized. Considering that patients are not always able to access applications such as physical therapy and hot pack in clinics due to the current conditions in our country, the cherry seed pillow may help to reduce the main symptom of pain. Patients can easily and safely use it at home by heating it in a microwave oven^14,16,25^.

The cherry seed cushion contributed positively to the patients’ level of being affected by FMS. Although the control group had better levels of being affected by fibromyalgia, we cannot ignore the effect of cherry seeds. Complementary and integrative therapies are recommended to reduce symptoms, relax people, and improve their quality of life^10,26,27^. The National Center for Complementary and Integrative Health (NCCIH) noted that although some research has been conducted on tai chi, yoga, mindfulness meditation, and biofeedback for fibromyalgia, there is not enough evidence in the current literature to support their conclusions^11^. In this context, our study will provide evidence.

## CONCLUSION

Cherry seed pillows were effective in reducing pain and symptom severity in patients with fibromyalgia.. Therefore, the cherry seed pillow can be used as an effective and safe complementary treatment method to reduce pain and fibromyalgia effects.

## Data Availability

The datasets generated and/or analyzed during the current study are available from the corresponding author upon reasonable request.
